# Identification of novel lactic acid bacteria with enhanced protective effects against influenza virus

**DOI:** 10.1371/journal.pone.0273604

**Published:** 2023-08-09

**Authors:** Atsushi Sugimoto, Tomoe Numaguchi, Ryota Chihama, Yuto Takenaka, Yuuki Sato

**Affiliations:** Niigata Research Laboratory, Mitsubishi Gas Chemical Company, Inc., Niigata, Japan; The University of Tokyo, JAPAN

## Abstract

Lactic acid bacteria (LAB) exert health-beneficial effects by regulating innate immunity in the intestinal tract. Due to growing health awareness, the demand for LAB and studies have focused on identifying beneficial LAB strains is increasing, especially those that stimulate innate immunity. In this study, the LAB strain D279 (NITE_BP-03645, *Latilactobacillus sakei*) was isolated from among 741 LAB strains that were analyzed for their ability to induce interleukin 12 (IL-12) production and was subsequently characterized. D279 induced the highest expression of IL-12 among the screened LABs. Furthermore, D279 significantly activated antiviral genes and preferentially induced interferon (IFN)λ expression *in vitro*, which plays a critical role in the epithelial tissue, thereby conferring strong anti-influenza potency without inflammation. However, it decreased the IFNα levels. The administration of pasteurized D279 to mice resulted in strong anti-influenza potency, with higher natural killer (NK) cell activity and a lower viral load in the lung than in the control. Importantly, none of the D279-administered mice were sacrificed during the viral infection tests. These results suggest that D279 administration confers beneficial effects by regulating innate immunity and that it may be relevant for commercial use in the future.

## Introduction

Lactic acid bacteria (LAB) have attracted considerable attention owing to growing health awareness regarding their beneficial effects. They act as intestinal regulators and have immunostimulatory and defense functions in the intestinal tract of hosts [1]. Several studies have been performed to elucidate the defense mechanisms of various LAB strains against viruses and pathogenic bacteria [2]. LAB with immunostimulating potency is in demand to prevent influenza and coronavirus infections. Therefore, identifying and characterizing biogenic LAB strains are critical in promoting human health; LAB also has applications in the food industry [3].

Immunostimulation by LAB is mediated by innate immunity [4–6]. In the intestinal tract, LAB is taken up by immune cells such as plasma dendritic cells (pDCs) or macrophages, or microfold cells on the Peyer’s patches, leading to the activation of innate immunity [7]. pDCs and macrophages recognize microorganism-associated molecular patterns (MAMP) based on pattern recognition receptors (PRRs), which localize to the immune cell surface and intracellular vesicles [8–10]. Toll-like receptors (TLRs) are the most well-characterized PRRs. To date, 13 types of TLRs have been identified in humans, each of which recognizes different MAMPs [11], indicating the versatility of the immune system. TLR1, TLR2, TLR4, TLR5, and TLR6 are expressed on the plasma membrane and recognize the membranes and lipoproteins of pathogens [11]. TLR3, TLR7, TLR8, and TLR9 are localized to the endosomes and recognize exogenous nucleic acids, such as non-methylated CpG-oligo DNA (CpG-ODN) and RNA molecules from pathogens [11].

TLRs promote the production of interleukins (ILs) and interferons (IFNs), resulting in the expression of antiviral factors that interfere with viral replication [12]. IL-12, a heterodimeric cytokine, is produced by macrophages or phagocytic cells and plays a primary role in driving innate immunity by triggering activated NK cells [13]. Additionally, innate immunity is orchestrated by three types of IFNs: Type I (IFNα, IFNβ, and others), Type II (IFNγ), and Type III (IFNλ). Among these, IFNα and IFNλ are particularly effective in protecting against viral infection [14, 15]. IFNα is receptive to cells throughout the body and immediately induces the expression of antiviral factors and inflammation [16]. In contrast, IFNλ, a recently discovered non-inflammatory IFN, mainly regulates epithelial cells that are in the front line of defense against infection, wherein the response is characterized by prolonged and strong induction of mucosal antiviral factors [15, 17]. Although there is an overlap between the antiviral genes induced by IFNα and IFNλ, reports indicate that IFNλ is more effective than IFNα in protecting cells from respiratory viruses [17, 18].

Several LABs are commercially available to prevent viral infection [19–24]. Most LAB that shows antiviral function act via the induction of inflammatory cytokines, frequently utilizing IFNα in TLR9-mediated pathways [25]. However, human health needs are diverse, and identifying beneficial LAB strains with strong immunostimulatory potential is challenging. Therefore, we screened 741 LAB to identify strains with immunostimulating potential by examining IL-12 production. IL-12 is an early response cytokine that acts against infections and activates NK cells to eliminate pathogens and infected cells that cause inflammation [26]. Thus, IL-12 is a good indicator for selecting beneficial LAB [27].

In this study, we aimed to screen LAB based on their IL-12 production capability, identify and characterize strains that induced the highest levels of IL-12, and determine whether the identified strain could activate innate immunity.

## Materials and methods

### Mice

We purchased 7.5-week-old male BALB/c mice from Charles River Laboratories Japan, Inc., and used for IL-12 quantification. Four-week-old female BALB/c Cr Slc mice from Japan SLC, Inc. were used for viral infection experiments because of the extensive information available on infection protocols and phenotyping methods [28]. For IL-12 quantification and influenza tests, 3 male BALB/c and 75 female BALB/c Cr Slc mice were used, respectively. Their body weights ranged from 13.2 to 16.3 g. The detailed experimental methods and breeding conditions are described below.

### Breeding conditions

Female BALB/c Cr Slc mice were pre-bred for 7 days. Mice were weighed on the day after procuring them and on the day the pre-breeding period was completed. The general growth conditions of the mice were observed daily, and those that showed no significant changes in body weight or general conditions were grouped for the experiments. The mice were bred in a room maintained at 19 to 24°C under 43 to 66% humidity with 12-/12-h light: dark conditions (light conditions: 0600–1800 h, dark conditions: 1800–0600 h) at Japan Bio Research Center Co., Ltd. (Gifu, Japan). During the pre-breeding period and after grouping, the mice were maintained in sterile plastic cages (W:175 mm × D:245 mm × H:125 mm) with floor mats (PaperClean Bedding; Japan SLC Inc., Shizuoka, Japan). The same conditions were used during the pre-breeding period and after grouping. Each cage was filled with environmental enrichment medium (nesting sheets, K3510; Animec, Tokyo, Japan) to ensure an optimal environment. During the pre-breeding period and after grouping, mice were bred individually. Feeders were changed during feeding, and the cages, water bottles, and environmental enrichment media were changed at least twice weekly. The rooms were cleaned and disinfected daily. During the pre-breeding period, the mice were fed powdered feed (MF; ORIENTAL YEAST Co., Ltd., Tokyo, Japan) within 9 months of manufacture *ad libitum*. After grouping, the animals were fed MF or MF mixed with 5% (w/w) of the test substance. Contaminants and bacterial counts in and the nutrient content of the feed were evaluated by Eurofins Food Testing Japan K.K. and ORIENTAL YEAST Co., Ltd. Drinking (tap) water was available *ad libitum*. Contaminants and bacteria in the drinking water were analyzed by Tohzai Chemical Industry Co., Ltd. The breeding conditions described were applied to all animal experiments performed in this study.

### Mouse groups

The mouse groups were stratified by body weight using a computer program (IBUKI, Japan Bioresearch Center Co., Ltd.), and the animals were randomly selected on the last day of the pre-breeding period (experiment 1: n = 8 per group (total 40 mice) for calculating survival rate, clinical score, weight, and amount of food consumption; experiment 2: n = 5 per group (total 25 mice) for calculating IgA, NK activity, and viral lung counts). The mean body weights and variances in each group were approximately equal. The remaining animals were anesthetized with isoflurane and euthanized by releasing blood from the abdominal aorta on the grouping day.

### Virus preparation

Influenza virus PR8 (A/PR/8/34 H1N1) and virus-containing media were prepared by the Japan Bioresearch Center Co., Ltd. (Gifu, Japan). Briefly, the cryopreserved virus stock solution was thawed, diluted 10^4^-fold in Dulbecco’s modified Eagle medium (DMEM; D5303; Sigma-Aldrich, St. Louis, MO, USA), and added to a culture flask containing MDCK cells. The culture flask was incubated for 1 h at 37°C in a CO_2_ incubator (MCO-170AICUV-PJ; Panasonic Healthcare, Osaka, Japan) for viral adsorption onto cells.

After removing the virus solution, DMEM with 0.25% trypsin (25300054, Thermo Fisher Scientific, Waltham, MA, USA) was added, and the cells were kept in a CO_2_ incubator until a cytopathic effect (CPE) was observed. The supernatant was centrifuged (4°C, 780 × *g*) in an AX-310 centrifuge (TOMY SEIKO, Tokyo, Japan) to remove foreign substances. The viral stock solution was frozen in an ultra-low temperature freezer until use.

The virus titer was determined using the Plaque assay. Briefly, the inoculated virus stock solution was prepared in DMEM in serial dilutions (10^3^-, 10^4^-, 10^5^-, and 10^6^-fold). The virus dilution (0.1 mL) was added to each well of a 12-well plate seeded with MDCK cells. The plates were shaken gently every 10 min to allow the cells to adsorb the virus for 1 h. The cells were then incubated for 1 h with equal amounts of minimum essential medium (MEM)-based medium and 1.6% (w/v) agarose solution. This medium was then incubated at room temperature until the agarose solidified. After that, the MDCK cells were incubated in a CO_2_ gas incubator for 2 days, and 1.5 mL of the MEM-based medium with neutral red was overlayed. Plaque count was calculated after incubation in the CO2 gas incubator overnight (n = 3). This virus stock was used for influenza infection.

### Influenza infection

The influenza virus PR8 (A/PR/8/34 H1N1) suspension at a density of 2 × 10^3^ PFU/mL was thawed, and 0.05 mL of virus-containing medium was dropped onto the nasal cavity of the mice (1.0 × 10^2^ PFU/mouse). The mice were fed MF *ad libitum* mixed with or without LAB powder at 0.5% (w/w) for 14 days before influenza infection. For experiment 1, the inoculation date was set as day 15, and this experiment was continued until day 23. Experiment 2 was continued until day 20. Optimal measures were taken to minimize the pain and stress to mice according to the humanitarian endpoints for the animal. All animal tests were performed, and the data were analyzed by the Japan Bioresearch Center Co., Ltd. The study was approved by the Committee of Animal Care of Japan Bioresearch Center Co., Ltd. (Test No. 410057).

### Mouse phenotyping and animal ethics

For all animal experiments, observation of mice and scoring of clinical outcomes were performed daily; the details are described in Table 1. Net body weight was measured once a week before the viral infection. After the viral infection, body weight measurements were performed daily. Food consumption was measured twice a week. The amount of food per feeder was measured using an electronic balance on the feeding day, and the remaining amount was measured on the next feeding day. The emaciation state was evaluated daily according to the humanitarian endpoint to calculate the survival rate, and the mice were immediately euthanized using isoflurane anesthesia by releasing blood from the abdominal aorta when they showed more than 20% loss of body weight or two or more areas with a clinical score of three (Table 1). For experiments 1 and 2, with respect to the survival rate, clinical score, weight, and food consumption, all mice survived before meeting the respective criteria for euthanasia. On day 20, 25 mice were euthanized using isoflurane before meeting the criteria for comparing the IgA levels, NK activity, and lung viral counts with those of the final euthanized group. The detailed methods are described below.

**Table 1:** Definition of clinical scores.

Score	Appearance			
	Eye	Coat	Behavior	Others
3	Eyelid adhesion	Poor hair coat	Decrease in spontaneous movements (no movement on contact)	Respiratory failure, emaciation, Supine position, lowering of body temperature
2	Loss of eyelid reflex (eyes open on contact, but no blink reflex)	Poor hair gloss, piloerection	Decrease in spontaneous movements (movement on contact)	Irregular respiration (severe), emaciation
1	Closed eyelid (eyes open on contact)	Slight piloerection	Response to stimulation	Irregular respiration (frequent or slow breathing)
0	Normal (open and bright eye)	Normal (Fine coat)	Normal	Normal (Regular respiration)

The clinical scores were based on the definitions provided in Table 1.

### IgA quantification

On day 20, the mice were anesthetized using isoflurane, and blood was collected using a syringe. Serum was obtained by centrifuging the blood at 2,000 × *g* for 15 min. IgA level was quantified using a mouse serum IgA ELISA Kit (ab157717; Abcam, Cambridge, UK) according to the manufacturer’s protocol.

### NK cell activity

NK cell activity was quantified using the LDH Cytotoxicity Detection Kit (MK401; Takara Bio, Shiga, Japan) according to the manufacturer’s protocol. Briefly, the mouse spleen was excised (with mice under anesthesia) on day 20 and squashed to obtain single cells in HBSS buffer (14025092; Thermo Fisher Scientific, Waltham, MA, USA). Erythrocytes were lysed by adding 2 mL of lysing solution (Ohtsuka, Japan). Mouse spleen cell cultures were diluted to 4 × 10^6^ cells/mL and co-incubated with FITC-labeled YAC-1 cells prepared at 1 × 10^5^ cells/mL.

### Lung viral counts

Viral load in the lungs was determined using a plaque assay. Briefly, on day 20, the mouse lungs were excised, sliced into small pieces, and homogenized in 2 mL of HBSS buffer (14025092; Thermo Fisher Scientific, Waltham, MA, USA) using a Physcotron homogenizer (Microtec Co., Ltd., Chiba, Japan). Homogenates were filtered using a cell strainer (431752; Corning, Corning, NY, USA) and centrifuged at 190 × *g* for 5 min to obtain the virus-containing supernatant. MDCK cells were incubated for 1 h with 0.1 mL of the virus supernatant to enable the influenza virus particles to adsorb to the cell surface. Next, an MEM-based medium containing 0.8% (w/v) agarose was overlaid onto the MDCK cells, which were grown for another 2 days. The cell sample was stained overnight with neutral red (140–00932; FUJIFILM Wako, Osaka, Japan) to observe the plaques and determine the viral count. Bronchoalveolar lavage fluid was not used in the study.

### Cell culture

HT-29 cells were obtained from the European Collection of Authenticated Cell Cultures, UK (ECACC). HT-29 cells were grown with 5% CO_2_ at 37°C in McCoy’s 5A medium (SH30270.01; Cytiva, Marlborough, MA, USA) supplemented with 10% fetal bovine serum (S0250; BioWest, Rue de la Caille, France) and 1% (v/v) penicillin and streptomycin (15140–122; Gibco, Grand Island, NY, USA). MDCK cells and YAC-1 cells were kindly provided by the Aichi Medical University School of Medicine Microbiology and Immunology Seminars.

### LAB strains and growth conditions

A total of 741 LAB strains were selected from a collection stored at –80°C. Each LAB was grown in MRS medium (63-6530-37; BD Difco, Sparks, MD, USA) at 30°C for 18 h. For IL-12 quantification and animal testing, LABs were grown and centrifuged at 2400 × *g* for 5 min. The bacterial pellet was washed with water thrice and sterilized at 80°C for 30 min. The LAB suspension was lyophilized (DF-05H-S; ULVAC, Kanagawa, Japan) at ˗40°C overnight to obtain a fine powder (pasteurized LAB). The LAB powder was used for IL-12 quantification at a final concentration of 20 μg/mL or mixed in the mouse feed at 0.5% (w/w).

### DNA extraction

Genomic DNA from the LAB strains was extracted using a NucleoBond AXG column and Buffer Set III (U0544A and U0603A; MACHEREY-NAGEL, Düren, Germany) according to the manufacturer’s protocol. The DNA concentration was measured using a SimpliNano spectrophotometer (29061712; Biochrom, Cambridge, UK) and used for lipofection.

### Lipofection

HT-29 cells were seeded in a 96-well plate at a density of 4 × 10^5^ cells/well, incubated overnight in DMEM supplemented with 10% (v/v) FBS, and then subjected to DNA lipofection. Lipofectamine2000 (11668–027; Thermo Fisher Scientific, Waltham, MA, USA) was used for DNA introduction, and the experiment was carried out according to the manufacturer’s protocol. When lipofection started, the medium was aspirated from the 96-well plate and 150 μL of solution containing 0.3% (v/v) lipofectamine 2000 and 5 μg/mL genome was added. Cells were further incubated overnight and used for ELISA and RNA extraction.

### Enzyme-linked immunosorbent assay (ELISA)

To quantify IL-12, the spleens of the male BALB/c mice were excised and gently squeezed to obtain cells for spleen cell culture. Cells were treated with 0.75% (w/v) ammonium chloride in 17 mM Tris-HCl (pH 7.7) to lyse the erythrocytes. After washing with 0.1% (w/v) BSA in PBS, cells were resuspended in RPMI 1640 medium (SH30027.01; Gibco, NY, USA) supplemented with 10% (v/v) bovine serum (S0250; BioWest, Rue de la Caille, France) and 1% (v/v) penicillin and streptomycin (15140–122; Gibco, NY, USA). The cells were cultured with 50 μM 2-mercaptoethanol in a 96-well plate at a density of 5 × 10^5^ cells/well and co-incubated with pasteurized LAB (20 μg/mL). After 48 h, the cellular supernatant was collected, and IL-12 quantification was performed. The supernatant was added to an immune plate (430341; Thermo Fisher Scientific, Waltham, MA, USA) coated with a monoclonal IL-12-B antibody (51004-R020; Sino Biological, Beijing, China). The plate was incubated at 4°C for 18 h, followed by blocking with 5% (w/v) skimmed milk containing 0.05% (w/v) Tween-20 at 37°C for 2 h. The plate was washed with PBS and 50 μL of the supernatant was added to the wells. The plate was then incubated at 37°C for 2 h. Next, 100 μL of biotinylated anti-IL-12 p35 (3456-6-250; MabtechAB, Nacka, Sweden) was added, and the plate was incubated at 37°C for 2 h. The plate was washed twice with PBS, 100 μL of streptavidin-HRP (RPN1231-2ML; Cytiva, Marlborough, MA, USA) was added to the wells, and the plate was incubated at room temperature for 1 h. After washing the cells with PBS, 100 μL of TMB substrate (5120–0047; SeraCare, Milford, MA, USA) was added to the wells, and the plate was incubated at room temperature for 1 h in the dark. After adding the TMB stop solution (5150–0020; SeraCare, Milford, MA, USA), the absorbance was evaluated at 450 nm using the microplate reader ARVO X3 (PerkinElmer, Shelton, CT, USA). Recombinant murine IL-12 p70 (210–12; PeproTech, Rocky Hill, NJ, USA) was used at appropriate dilutions as the standard.

For IFNα and IFNλ quantification, HT-29 cells were cultured in 96 well plates at a density of 4 × 10^5^ cells/well for 24 h. Next, genomic DNA from LAB or Poly (I: C) (4287/10; R&D Systems, Minneapolis, MN, USA) was transfected into the cells at a final concentration of 5 and 1 μg/mL using Lipofectamine 2000 (11668027; Thermo Fisher Scientific, Waltham, MA, USA), respectively, according to the manufacturer’s protocol. The cells were further cultured for 24 h, and the supernatant was used to quantify IFN using the VeriKine-HS Interferon α All Subtype TCM ELISA Kit (41135–1; PBL Assay Science, Piscataway Township, NJ, USA) and the Human IL-29/IL-28B (IFNλ1/3) DuoSet ELISA Kit (DY1589; R&D Systems, Minneapolis, MN, USA) according to the manufacturer’s protocols.

### Electron microscopy

The phenotype of D279 cells was evaluated to determine whether the cells were round or rod-shaped using electron microscopy. D279 cells were grown in MRS medium (63-6530-37; BD Difco, Sparks, MD, USA) overnight and treated with the ionic liquid HILEM IL1000 (Hitachi High-Tech, Tokyo, Japan) at a ratio of 1:1 for 1 h at room temperature. The mixture was dropped onto filter paper and dried until the water evaporated. After fixing the filter paper on the SEM specimen table, the sample was examined at 0.5 kV using FE-SEM SU9000 (Hitachi High-Tech, Tokyo, Japan).

### Phylogenetic analysis

As D279 was not classified, the 16S rDNA sequence of D279 was analyzed using the ABI PRISM 3500xL Genetic Analyzer System (Thermo Fisher Scientific, Waltham, MA, USA), and a phylogenetic tree was created using the maximum-likelihood or neighbor-joining methods.

### qRT-PCR

Total RNA was extracted from HT-29 cells using the CellAmp Direct RNA Prep Kit for RT-PCR (3732; Takara Bio, Shiga, Japan) after introducing genomic DNA or Poly (I:C). qRT-PCR was performed using the One Step TB Green PrimeScript PLUS RT-PCR Kit (RR096A; Takara Bio, Shiga, Japan) and a Thermal Cycler Dice Real Time System TP800 (Takara Bio, Shiga, Japan). *GAPDH* was used as an internal control. Primers were synthesized by Integrated DNA Technologies (Coralville, IA, USA). The specific primer sequences were as follows: *GAPDH* (forward: 5ʹ-GAAGGTGAAGGTCGGAGTC-3ʹ, reverse: 5ʹ-GAAGATGGTGATGGGATTTC-3ʹ), *MX1* (forward: 5ʹ-ACAGGACCATCGGAATCTTG-3ʹ, reverse: 5ʹ-CCCTTCTTCAGGTGGAACAC-3ʹ), and *OAS1* (forward: 5ʹ-TGTCCAAGGTGGTAAAGGGTG-3ʹ, reverse: 5ʹ-CCGGCGATTTAACTGATCCTG-3ʹ).

### Statistical analysis

**Fig 1:**
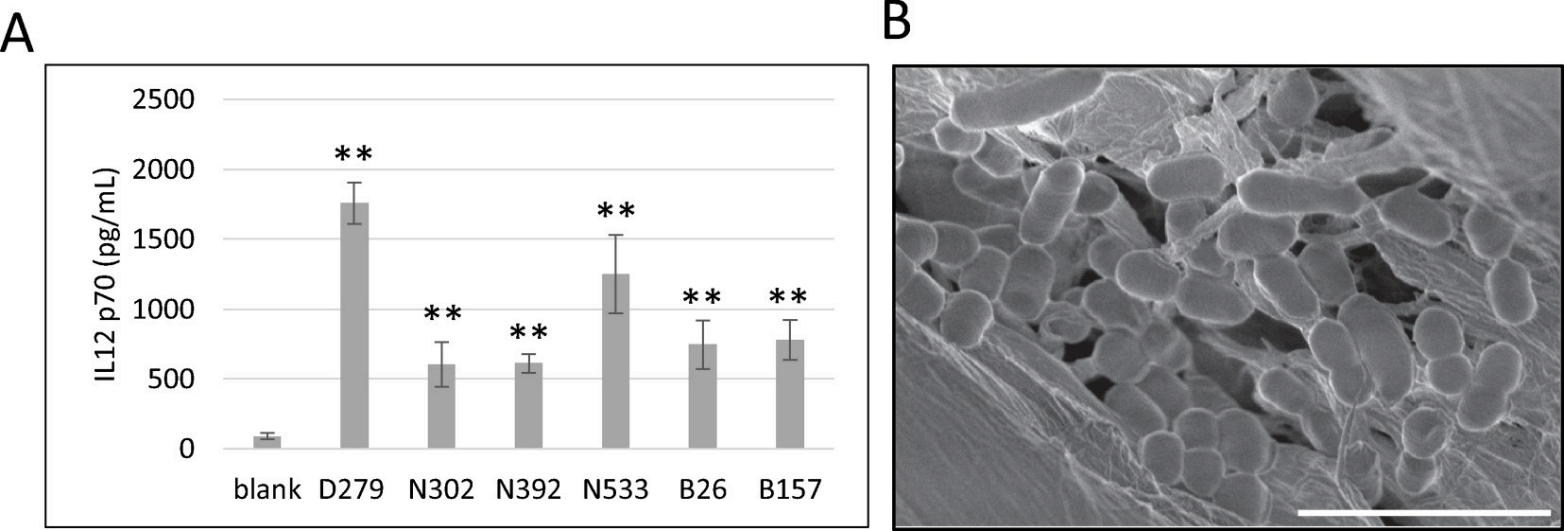
Screening the functional LAB with immunostimulatory activity. (A) IL-12 activity. Cells from the spleen of mice were cultured overnight with pasteurized LABs. The level of IL-12 p70 produced was quantified using ELISA. The top six strains (> 600 pg/mL) are shown in the graph. The values are presented as mean ± S.E. (n = 3). Asterisks indicate a statistically significant difference compared with the mock control analyzed using Student’s *t*-test (**P < 0.01). (B) Electron microscopy image of D279. Scale bar = 5 μm.

**Fig 2:**
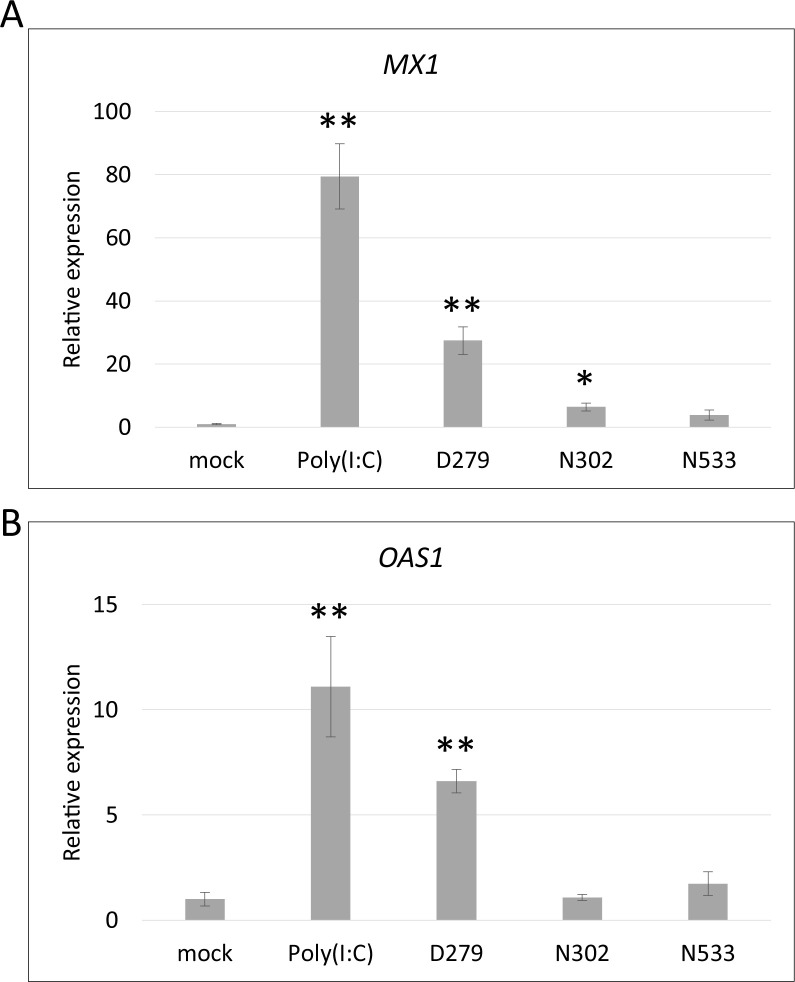
Expression analysis of antiviral genes using quantitative RT-PCR. qRT-PCR analysis of the antiviral genes. Twenty-four hours after introducing the LAB genomes using lipofection, total RNA was extracted from HT-29 cells and subjected to qRT-PCR. Mock (non-treated), Poly(I:C) (Poly(I:C) treated), and LAB (LAB genome introduced) were analyzed. *Gapdh* was used as an internal control, and the results are shown relative to the expression of the mock control. Values are presented as mean ± S.E. (n = 4). Asterisks indicate a statistically significant difference compared with the mock control analyzed using Student’s *t*-test (*P < 0.05, **P < 0.01). (A) Expression of *MX1*. (B) Expression of *OAS1*.

**Fig 3:**
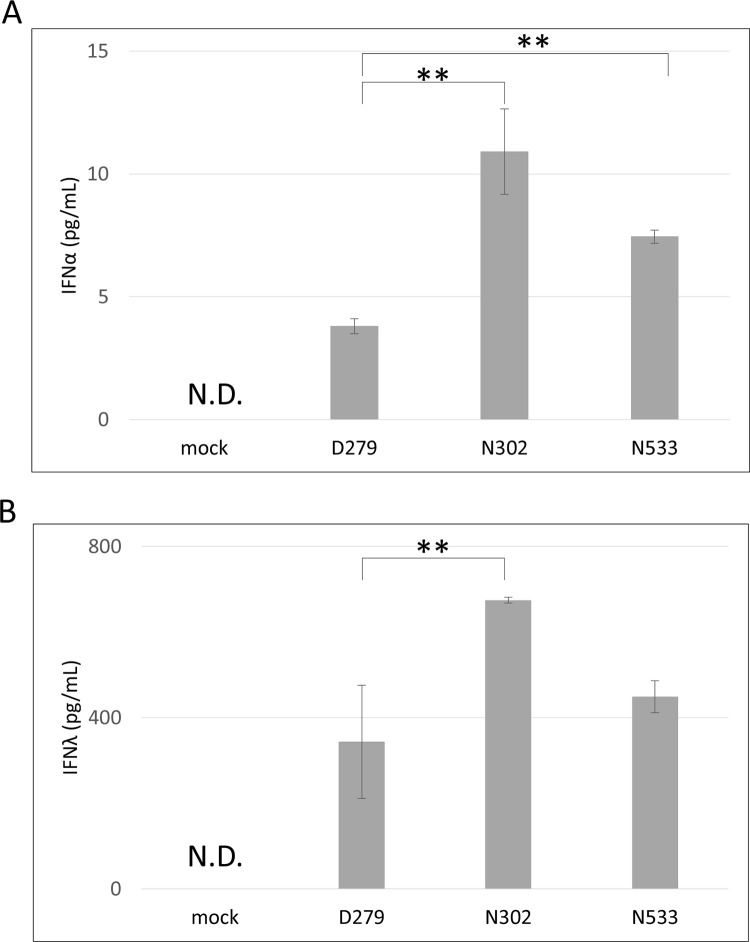
IFN induction capability of D279. LAB genomes were introduced into HT-29 cells using lipofection. After 24 h, the medium was collected and subjected to an enzyme-linked immunosorbent assay. Cultures were performed in triplicate to quantify IFNs in each experiment. Values are presented as mean ± S.E. (n = 3). N.D. indicates not detected. Asterisks indicate a statistically significant difference compared with D279 analyzed using Student’s *t*-test (**P < 0.01). (A) All IFNα subtypes. (B) All IFNλ subtypes.

**Fig 4:**
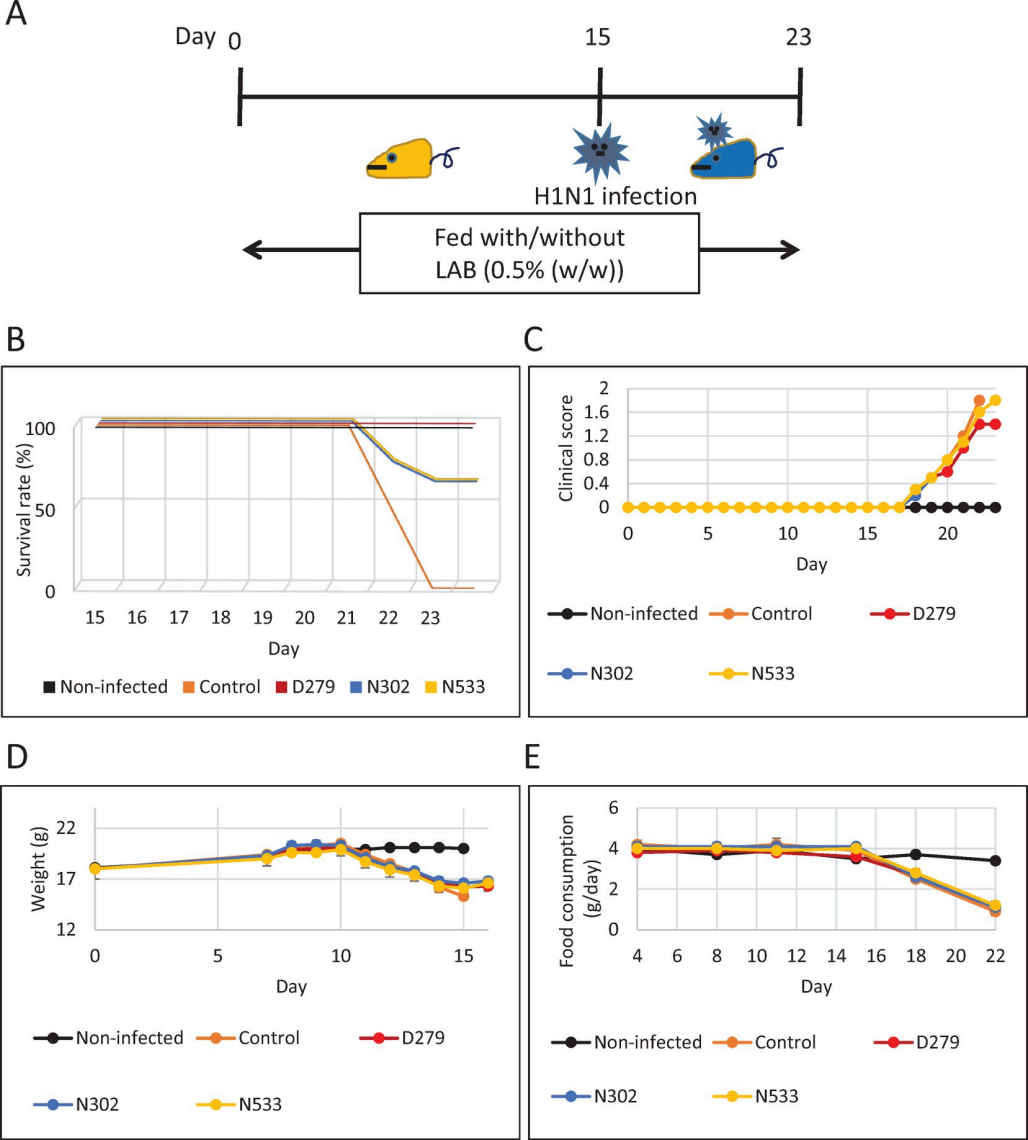
Effect of D279 during H1N1 infection testing. Experiments with a mouse influenza model were performed to investigate the immunostimulatory function of LAB. Mice were classified into non-infected, control (infected, normal feeding), and LAB-fed (infected, fed LAB) groups (n = 8 each). The clinical scores, survival rates, feeding amount, and body weight were measured daily until day 23. (A) Experimental design for the H1N1 infection test. Mice were administered food with/without LAB for 14 days before infection. On day 15, the mice were infected by dropping 50 μL of influenza virus-containing water onto the nasal cavity (1.0 × 10^2^ PFU/mouse). (B) Survival rates. In each group, the survival rate was monitored daily. (C) Clinical scores. From day 0 to day 23, phenotypes were observed and scored according to the severity of the body condition. A higher score indicates a more severe condition. The mice in the control group were sacrificed on day 22 per the humanitarian endpoint. (D) Body weight. During the H1N1 infection experiment, the body weight of the surviving mice was measured daily. The values are presented as mean ± S.E. (E) Food consumption. Changes in food consumption were measured twice a week. On the day of feeding, the amount of food in each feeding container was weighed using an electronic balance, and the remaining amount was measured on the next feeding day. Values are presented as mean ± S.E.

**Fig 5:**
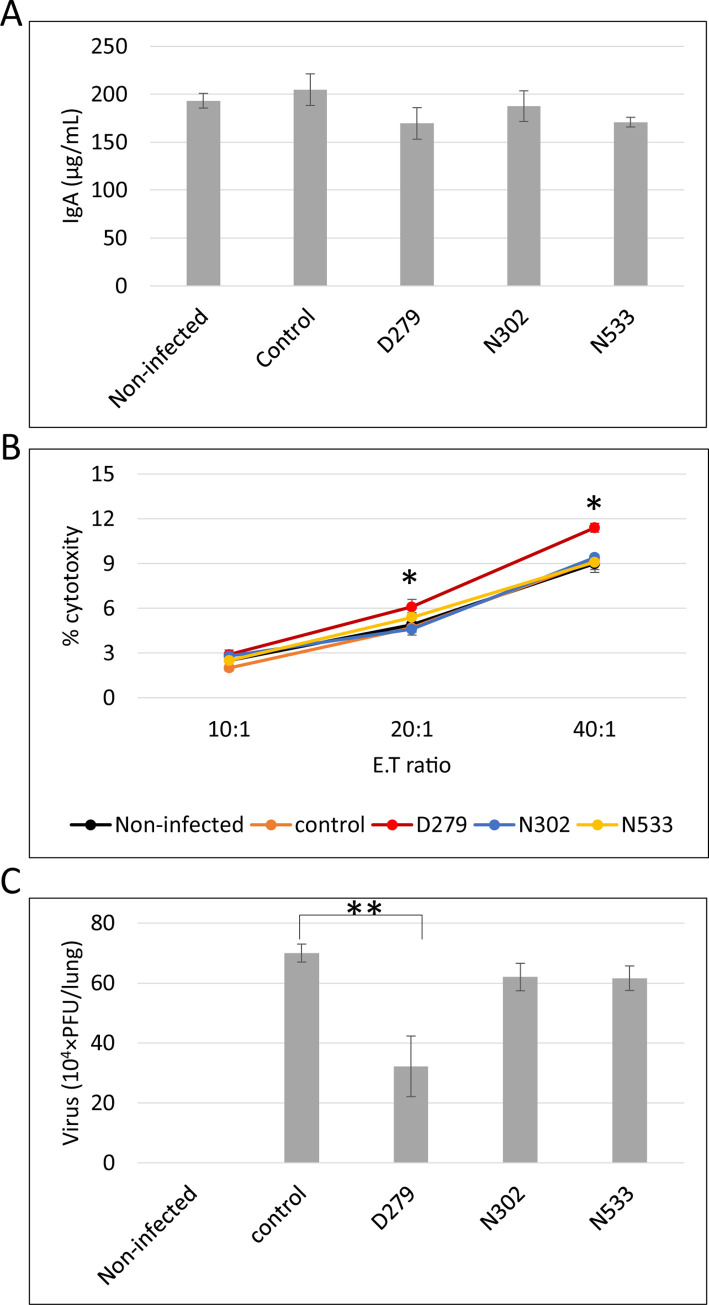
Immunostimulatory activity of D279. The asterisk shows a significant difference using Wilcoxon’s test (*P < 0.05, **P < 0.01) compared to the control samples. The bar shows mean ± S.E. (n = 5). (A) IgA quantification. Five days after viral inoculation, serum was obtained from the blood of mice and used to quantify the IgA level using ELISA. (B) NK activity. After euthanasia, mouse spleens were excised to obtain single-cell suspensions. NK activity was evaluated. (C) The viral load in the lung. The lungs of mice were excised after euthanasia, and lung virus titers were calculated using the plaque assay.

The data presented in Figs 1–3 were analyzed using the two-tailed paired Student’s *t*-test in Microsoft Excel. The data presented in Figs 4 and 5, and S1 Fig were evaluated as follows: for body weight, general condition score, food intake, viral number in the lungs, NK activity, and IgA levels, the mean and standard error for each group were calculated. The average daily food intake was calculated from the feeding day to the residual measurement day. Wilcoxon’s rank-sum test was used to evaluate the general condition score. Survival analysis was performed using the log-rank test, and the analysis was adjusted to account for the multiplicity of between-group comparisons. After analyzing the equivalence of variances with the F-test, body weight measurements were evaluated using Student’s *t*-test when variances were equal and Aspin-Welch’s *t*-test when variances were unequal. Statistical analysis was performed using a commercial statistical program, SAS (SAS Institute Japan, Tokyo, Japan).

## Results

### Identification of LAB with high potential to activate innate immunity

A total of 741 LAB strains were screened. The immunostimulatory ability of the LAB was evaluated based on IL-12 p70 production in splenocyte culture. IL-12 is a critical cytokine that plays a central role in activating innate immunity and is used as a major index in LAB screening as it can activate NK cells [26, 29]. Here, for screening, pasteurized LAB at a concentration of 20 μg/mL was added to the splenocyte culture as pasteurized LAB is stable and effectively stimulates innate immunity [30]. The level of IL-12 in the culture was quantified using ELISA. Six of the 741 strains induced significant production of IL-12 (>600 pg/mL) (Fig 1A). Among these strains, D279 induced the highest levels of IL-12 (Fig 1A). D279 was identified as *Latilactobacillus sakei* based on the phylogenetic analysis using the 16S rDNA sequence (S1 Fig), and showed a rod-shaped phenotype (Fig 1B). D279 strain was used in the subsequent experiments. For comparison, N533 (NITE_BP-03647, *Pediococcus pentosaceus*) and N302 (NITE_BP-03646, *Lactiplantibacillus plantarum*) strains were used for further analysis, as N533 and N302 induced the second-highest and lowest production of IL-12, respectively.

### Quantitative RT-PCR analysis of antiviral genes

The expression levels of the antiviral genes MX dynamin-like GTPase1 (*MX1*) and 2ʹ,5ʹ-oligoadenylate synthase1 (*OAS1*) were analyzed *in vitro* as indicators of immunostimulation because these genes inhibit viral gene replication for obtaining additional evidence for the immunostimulatory function of D279 [31, 32]. HT-29 cells were used as a gut epithelial model to evaluate immunostimulatory potency [33]. HT-29 cells were cultured overnight in 96-well plates, and the genomes of D279, N302, and N533 were introduced by lipofection. Total RNA was extracted after 24 h of culture and analyzed using qRT-PCR. The expression of *MX1* and *OAS1* was highly induced in genomic DNA-introduced cells; however, the D279 genome presented the highest expression of these genes among the LAB analyzed (Fig 2A and 2B). The result indicates that D279 possesses strong immunostimulatory activity *in vitro*.

### IFN production *in vitro*

To investigate the mode of action of D279, Type I and III IFN production was analyzed *in vitro* because of their pivotal role in viral defense. These IFNs are known to be induced by the LAB genome via the TLR9 pathway and act as key factors in inhibiting viral replication by inducing interferon-stimulating genes [14, 15]. Here, HT-29 cells were grown in a 96-well plate for 24 h, and the LAB genomes were introduced using lipofection. N302 and N533 significantly induced IFNα and IFNλ production compared to the mock control (Fig 3A and 3B). In HT-29 cells transfected with the D279 genome, IFNα was produced, but the levels were significantly lower than those induced by N302 and N533 (Fig 3A), whereas IFNλ was induced at levels similar to those by N533. The ratio of IFNα to IFNλ was 1:90 for D279, whereas that for N302 and N533 was 1:60, indicating that D279 preferentially induces Type III IFN.

### Influenza infection test

To test the biogenic effects of D279, a mouse influenza (H1N1) infection model was used. Mice were fed with or without LAB powder mixed at 0.5% (w/w) *ad libitum* for 14 days before influenza virus infection (Fig 4A). The mice were divided equally into three groups (n = 8 per group): non-infected (no infection, normal feeding), control (infected, normal feeding), and LAB-fed (infected, fed D279, N302, and N533). On day 15, the mice were infected with the influenza virus by dropping influenza virus-containing medium onto the nasal cavity (1.0 × 10^2^ PFU/mouse) (Fig 4A). The test was continued until day 23 (Fig 4A). In the control group, half the mice were euthanized on day 21, and the other half were euthanized on day 22 according to humanitarian endpoints; all mice survived in the non-infected group (Fig 4B). N302 and N533 showed antiviral function, as the survival rate improved to 63% on day 23. Importantly, the D279-administered group showed a 100% survival rate during the test period (Fig 4B), indicating greater beneficial effects than the other LAB strains.

Additionally, mouse phenotypes were observed daily and evaluated for clinical scores (Fig 4C, Table 1). The score increased when the symptoms worsened after infection (Table 1). The D279-administered group showed a slower increase in the clinical score than the other groups (Fig 4C), whereas body weight and food intake continued to decrease throughout the study period (Fig 4D and 4E). This finding suggests that the higher survival rate of the D279-administered group is related to the immunostimulatory activity of D279. Overall, these results indicate that the oral administration of D279 before influenza virus infection effectively prevents a viral invasion.

### Mechanism of immunostimulation by D279

*In vivo* experiments demonstrated that D279 alleviated influenza symptoms. The degree of activation of humoral and cellular immunity was examined to determine the higher survival rate of mice in the D279-administered group. The level of IgA was quantified; however, its production did not differ among the groups (Fig 5A), indicating that D279 did not stimulate humoral immunity. Next, NK activity was quantified by evaluating the number of NK cells that attacked FITC-labeled cancer cells *in vitro*. The LAB-administered groups showed higher NK activity than the non-infected and control groups; however, in the D279-administered group, the NK activity was significantly higher than that in the other LAB-administered groups (Fig 5B). Furthermore, viral load in the lungs was quantified using a plaque assay. The D279-administered group showed a significant reduction in viral counts (Fig 5C). These findings indicate that D279 stimulates cellular but not humoral immunity *in vivo*.

## Discussion

In this study, D279 was identified as a useful strain that conferred a 100% survival rate in influenza virus infection tests in mice. D279 was isolated from among 741 strains of IL-12-producing LAB. D279 was identified as *L*. *sakei*, and its immunostimulatory activity has not been evaluated. Several strains of the genus *L*. *sakei* have been isolated from processed meat, fish, and vegetable products and are traditionally used as fermentation starters [34, 35]. The type strain of *L*. *sakei* ATCC15521 (subsp. *sakei*) was isolated more than 80 years ago and has been shown to have immunostimulatory functions [36]. D279 is a plant *Lactobacillus* that was originally isolated from the Japanese pickles *Aka-kabu zuke* (a red turnip pickled with LAB and with/without salts). The pickle is a traditional food that has long been consumed during winter when influenza and other respiratory viruses are present. It is interesting to note the traditional methods people adopt in their daily diet to prevent viral infection. In addition, some species of the genus *L*. *sakei* reportedly activate mucosal immunity [37–39]. Thus, *L*. *sakei* appears to be highly beneficial for human health.

Here, the oral administration of D279 to mice substantially improved their survival rate after influenza infection, and none of the mice died during the experimental period, whereas the other LAB-administered groups showed a reduced survival rate. The improvement in survival rates resulted from the immunostimulatory effect of D279, as dietary intake did not change between groups with or without D279 administration (Fig 4E). Several LAB strains have antiviral effects [40, 41]. For example, orally administered *Lactobacillus pentosus* ONRICb240, *Lactobacillus delbrueckii* OLL1073R-1, *Lactobacillus casei* strain Shirota, *Lactobacillus crispatus* KT-11, and *Lactobacillus pentosus* strain S-PT84 exerted antiviral effects in mice, via the activation of macrophages, NK cells, IgA, Type I IFN, TNFα, and IL-6 after Th1 polarization [42–46]. The macrophage-NK cell pathway is frequently activated by LAB and is a key barrier against viral infection. *Lactococcus lactis* JCM5805 activates innate immunity by directly stimulating pDCs to promote type I IFN production [19]. D279 administration increased NK cell activity but not IgA production during the influenza infection test (Fig 5A and 5C), and D279 may be taken up by macrophages, which may stimulate the production of IL-12 and activation of NK cells. IgA production requires Type I IFN from pDCs, B cell activating factor from the TNF family (BAFF), and a proliferation-inducing ligand (APRIL) to stimulate B cells [47, 48]. As D279 does not significantly induce IgA production, uptake by pDCs and their activation may not occur. Thus, oral administration of D279 directly eliminated influenza viruses by driving cellular immunity, including producing macrophages and NK cells.

Importantly, D279 stimulates IFN production *in vitro*. The ratio of IFNα:IFNλ was 1:90 for D279, whereas that for N302 and N533 was 1:60. The result indicates that D279 preferentially induces IFNλ to activate innate immunity. IFNα and IFNλ induce the expression of the same subset of antiviral genes; however, the distinct role of IFNλ has been identified recently. Davidson et al. demonstrated the effectiveness of IFNλ, rather than IFNα, against respiratory viruses, and their results showed that the administration of IFNα to influenza virus-infected cells or mice reduced the viral load, although inflammatory stress resulted in necrosis or death [17]. However, IFNλ administration effectively decreased viral load and resulted in a better survival rate than IFNα treatment. A dramatic improvement in the survival of the D279-administered group may partially result from reduced inflammatory immune activation. However, one limitation of this study is that we did not obtain enough data on cytokine production, including IL-12, IFNα, and IFNλ *in vivo*, because of the limited number of mice for in-depth analysis. Further *in vivo* studies are required to quantify IFN production, and that of other inflammatory and noninflammatory cytokines.

In future studies, it is important to elucidate the immunostimulatory mechanism of D279, determine the mechanism through which D279 regulates IFNs or antiviral factors, and clarify how immune signals in the intestinal tract are delivered to the lungs to function. The components of D279 that are activated and how they affect innate immunity to result in a 100% survival rate are unclear. Here, D279 exhibited antiviral effects, making it a valuable strain for commercial use. Importantly, D279 can be used as a food additive or supplement for health maintenance.

## Supporting information

S1 Fig Classification of D279. Maximum-likelihood phylogenetic tree of D279 with related strains. The 16S rDNA sequence was aligned, and bootstrap analysis was performed with 1000 replicates. The scale bar indicates 0.01 substitutions per nucleotide position.
(TIF)

S1 Data (XLSX)
