# Supplementary figures and images for: Identification of novel lactic acid bacteria with enhanced protective effects against influenza virus
Source: PLoS One. 2023 Aug 9;18(8):e0273604. doi: 10.1371/journal.pone.0273604 (PMC10411811; doi:10.1371/journal.pone.0273604)

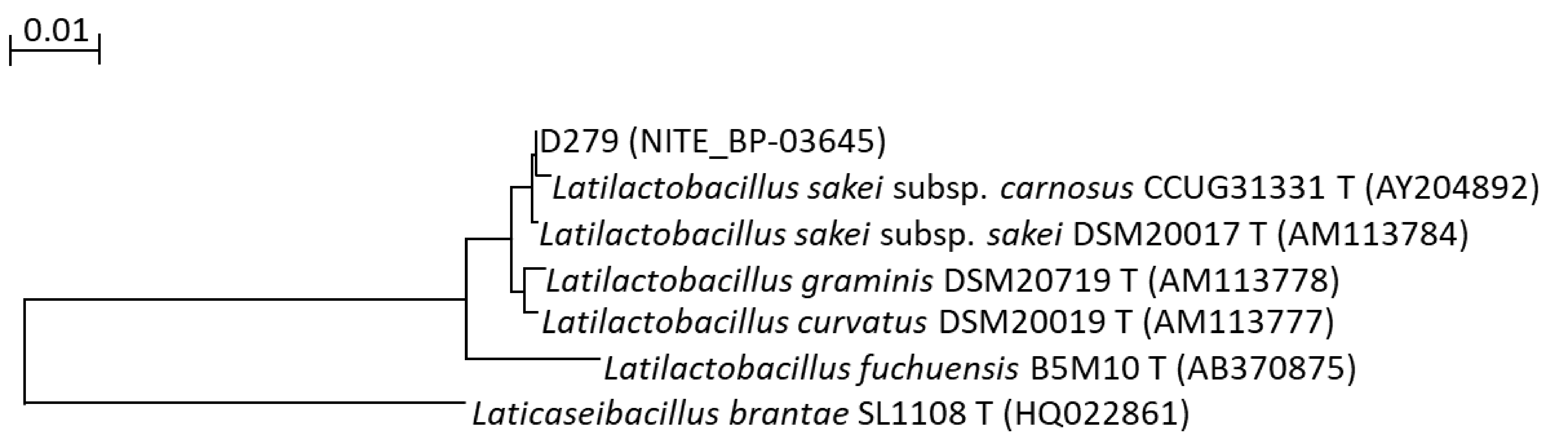

Supplement: S1 Fig — Maximum-likelihood phylogenetic tree of D279 with related strains. The 16S rDNA sequence was aligned, and bootstrap analysis was performed with 1000 replicates. The scale bar indicates 0.01 substitutions per nucleotide position. (TIF) [file pone.0273604.s001.tif]
